# Managing Grief of Bereaved Families During the COVID-19 Pandemic in Japan

**DOI:** 10.3389/fpsyt.2021.637237

**Published:** 2021-06-04

**Authors:** Yoko Matsuda, Yoshitake Takebayashi, Satomi Nakajima, Masaya Ito

**Affiliations:** ^1^National Center of Cognitive-Behavior Therapy and Research, National Center of Neurology and Psychiatry, Kodaira, Japan; ^2^Department Health Risk Communication, School of Medicine, Fukushima Medical University, Fukushima, Japan; ^3^Department of Human Sciences, Faculty of Human Sciences, Musashino University, Koto-ku, Japan

**Keywords:** COVID-19, prolonged grief, ambiguous loss, mourning process, bereavement

## Abstract

This commentary discussed the psychological issues related to bereavement in the wake of the COVID-19 pandemic. Specifically, we addressed two aspects in the context of Japanese culture. The first relates to the psychological distress of members of the bereaved family who could not visit their loved ones who had COVID-19 before or after their death. The second relates to the bereavement experience of those who were unable to be with their loved ones when the end came, even though they did not have COVID-19, because of restrictions on visiting hospitals. We seek to focus on the need for a support system for bereaved families to help them through the grieving process, and discuss end-of-life care in such circumstances, and in the post-COVID-19 era, as in current day Japan.

## Introduction

The number of deaths worldwide due to the COVID-19 pandemic has exceeded 3 million as of April 20, 2021 (1). In this pandemic, the grief response may become more complex and prolonged, leading to psychological problems among people. During the same period, the pandemic caused 9,629 deaths in Japan, a 2% mortality rate (1). Thus, the number of COVID-19-related deaths has been relatively lower in Japan than in the Western countries, where the infection has exploded (2). As the number of deaths due to the pandemic increases, there is growing focus on the suffering of the bereaved families and their support systems in the wake of the COVID-19 pandemic. Pre and post-face-to-face family contact with loved ones is limited in in order to prevent infection (3). The suddenness and unpredictability of bereavement due to COVID-19 makes it challenging to establish advance care planning, a key component of effective terminal care (4). Bereavement due to COVID-19 infection may also interfere with the adaptive mourning process in terms of disruption of social norms, rituals, and mourning practices for death, as seen during past epidemics of infectious diseases (5). Because of these peculiarities, there is concern that bereavement due to coronavirus infections may increase psychological risks, such as complicated grief and depression (5, 6). For this reason, safer funeral practices have been explored in infectious disease pandemic disasters through the modification of funeral rites and the introduction of traditional ritual techniques specific to each culture (6, 7).

In the early stages of the pandemic, public awareness regarding COVID-19 deaths was low because there was no rapid increase in the number of deaths in Japan. However, public awareness increased considerably following the mass media broadcasts of the deaths of two celebrities from COVID-19–a popular comedian and a famous actress–in March and April 2020, respectively. Around the same time, there were infections among funeral workers, which raised public concern about conducting funerals in these difficult times. It became apparent that bereavement during the COVID-19 pandemic was different from that caused by other types of death in Japan. We addressed two issues related to bereavement. First, family members of persons succumbing to COVID-19 were not allowed to bid goodbye to the deceased before death or see them face-to-face even after death. Second, the impact on end-of-life care and bereavement due to restrictions on visits to hospitalized patients, which had significant ramifications given the fact that a majority of people die in hospitals in Japan (8). We shall discuss efforts to address these issues in Japan.

## Potential Psychological Concern Regarding Bereavement Due to COVID-19

In early April 2020, during the initial stages of the pandemic, a statement regarding its psychological impact was released by the Chairman of the Disaster Preparedness and Disaster Response Committee of the Japanese Society for Traumatic Stress Studies. The statement pointed out that bereavement due to COVID-19 could lead to psychological problems such as prolonged grief and ambiguous loss, one which is without closure or clear understanding of why it occurred (9). In order to prevent the spread of COVID-19 in Japan, it was recommended, in principle, that visiting a person at the end of life with COVID-19 infection should be prohibited (10). Not being present at the death of patients with COVID-19 infection impedes the usual farewell rituals conducted for the deceased by the bereaved. For example, touching the deceased's body helps those bereaved to realize that their loved one has actually died. Not being present at the end of a person's life and not being allowed to touch the body potentially interferes with the mourning process and increases the risk of prolonged grief (9). In the Netherlands it was noted that grief levels were higher among people bereaved due to COVID-19 than those who had lost loved ones due to natural causes (11). Traumatic bereavement is more likely to occur when the death of a loved one is sudden or unnatural, such as when a loved one's body is damaged (12). It is accompanied by regret, anger, and guilt over death (For example, could the end have been prevented? Is the loved one suffering? Was death or dying unjust?) (12). Traumatic bereavement is a risk factor for PTSD and depression, as well as prolonged and complicated grief (13). These symptoms after bereavement often co-occur and share common features, but many epidemiological studies support their distinctiveness (14, 15). Since bereavement due to COVID-19 shares the common feature of traumatic bereavement (6), systematic psychosocial support is needed. Psychological therapies, such as cognitive-behavioral therapy, optimized for each symptom, have been shown to improve PTSD, grief, and depression after bereavement (16, 17).

Although Japan has yet to report any empirical studies on grief after bereavement due to COVID-19, considering its cultural practices regarding funerals, it is reasonable to expect increased rates of prolonged grief. In Japan, it is customary to hold a funeral that is widely attended by family members, locals, or business partners. Such bereavement ceremonies are essential to facilitate the grieving process of the bereaved by allowing them to share their feelings and memories of the deceased. Traditional Japanese funeral ceremonies include sharing meals and alcohol with all the people who attend. However, most funerals have now been restricted in Japan, following instances of people becoming infected with COVID-19 after attending funerals. Being in close physical proximity with friends or others may produce feel-good hormones such as oxytocin, dopamine, and serotonin (18). When they are not physically present to say goodbye and grieve with a loved one, they may be more likely to experience a sense of ambiguous loss (18). An ambiguous loss is an indefinite loss that persists without resolution or closure, such as when a loved one is missing (e.g., kidnapped or swept away by a tsunami and never found) or physically present but psychologically absent (e.g., the former personality is still intact due to dementia) (19). Ambiguous loss differs from ordinary bereavement in that there is no definitive information or finality (19).

In Japan, guidelines regarding COVID-19 patients have been in place right from the beginning of the pandemic. For example, the number of visits to critically ill patients should be limited. Existing restrictions relate to both end-of-life care and contact with the body after death. However, there is the need to take care of family feelings at each stage, which highlights the usefulness of communication through social networking services and online tools in the COVID-19 Nursing Practice Guide for Critically Ill Patients, Version 1 (published in April 2020) (20). In response to such recommendations, efforts at the grassroots level are underway to encourage communication between patients and their families through online communication tools such as videophones. For example, a physician has launched a crowdfunding campaign to purchase tablets in hospitals to facilitate online communication between critically infected patients and their families. He reached his goal in just half a day (21), and by the end of the campaign, he had raised more than five times his goal of more than 16 million yen, enabling distribution of the tablets to approximately 80 facilities (22). Similar efforts–making videoconferencing possible in end-of-life care settings, when patients cannot see their families face-to-face because of the pandemic (23)–are expected to alleviate patients' suffering in a way that medical personnel by themselves cannot. There is also a growing focus on comforting patients in their dying days, with families asking medical personnel to show the patients their favorite pictures and play their favorite music (24).

From the perspective of preventing infection during transportation and cremating the bodies of those who have succumbed to COVID-19, the Japan Medical Association's Implementation Manual (6th edition) (25) requires that crematorium workers and mourners do not touch the body. Under these circumstances, to arrange the farewell ceremony close to the conventional one, flowers and photographs are placed on top of the coffin (which usually mourners set inside the coffin). In case there are restrictions on the number of people who can be present at the funeral, the cremation service provider can take pictures of the deceased before the funeral and show them to the family later. The family could also ask the service provider to place photographs, flowers, and other items related to the deceased on the coffin. Such acts at the funeral ceremony perhaps reflect the bereaved family members' sentiments that they are not leaving their loved ones alone at the time of their death. In addition, there is a custom of wiping and cleansing the body and applying makeup on the face after death (so-called angel care), which nurses generally perform when patients die in Japan (26). However, Version 2 of the COVID-19 Nursing Practice Guide for Critically Ill Patients published in July 2020 points out the importance of family members' participation in angel care in terms of grief care (26). The guideline recommends explaining the risk of infection to family members. If they still wish to participate in angel care, one recommendation is that they take the same preventive measures as do the medical personnel like wearing protective clothing, and touching a safe body area where they are not exposed to bodily fluids. Continuous efforts are needed to make mourning rituals safer with technology, and changes in funeral practices acceptable in each local culture (19).

## The Impact of Restricted End-of-Life Care Visits for Non-COVID-19 Inpatients

Japan has been a super-aged society since 2007. In 2019, 28.4% of the population were 65 years and above and 4.7% were 85 years or older (27). In the 1950s, more than 80% of people died at home in Japan (28). In 2017, more than 80% of patients died in hospitals or institutions (8), despite nearly 70% of them wanting to die at home (29). Fewer people die at home because of the increasing trend toward nuclear families, which has led to a decline in family relationships (28). Many older relatives are moved into homes for the elderly because it may be a burden on family members and others who care for them (29). It is necessary to improve home medical care in present-day Japan to achieve end-of-life care at home, but only 5% of all medical institutions could support it in 2014 (8). For these reasons, many patients choose to receive end-of-life care for diseases other than COVID-19 in a medical facility or palliative care. However, many hospitals now restrict visits to non-COVID-19 inpatients—a necessary and natural measure—to prevent nosocomial infections (infections caused by pathogens in the hospital). There is a concern that these measures will result in a situation where terminally ill, non-COVID-19 patients, will not be provided with adequate end-of-life care. One of the risk factors for prolonged grief and PTSD after the death of a COVID-19 patient is the bereaved family's inability to say goodbye to the deceased. Since family members of non-COVID-19 inpatients have restricted visitation, the same psychopathological risk can be assumed for them (3). In light of the philosophy of palliative care, it is desirable for the psychological health of patients and their families to spend time with each other so that the patients are taken care of at the end of their lives, by their own. Therefore, there has been a move to provide a flexible response so that patients can be involved in end-of-life care while taking measures to prevent infection.

In response to this situation, the Japanese Society for Palliative Medicine has suggested in a pamphlet that families should consider caring for patients at home (30). In one such case, based on a nurse's suggestion, a leukemia patient who was prepared to die in the hospital could go home and spend the next 10 days with his family (31). In some areas, the number of individuals switching to end-of-life care at home has nearly doubled compared to previous years (32). Cases of end-of-life care at home have also been reported in the UK and Portugal (33, 34). In Portugal, most families encourage terminally ill patients to stay at home for an extended period (34).

Although, in some cases, family members were unable to visit their hospitalized relatives freely during the COVID-19 pandemic, they asked individuals, called end-of-life caregivers, from some organizations such as hospices, to provide end-of-life care for terminally ill patients living alone in Japan (35). Conversely, some physicians involved in palliative care have expressed concern about suggesting end-of-life care at home. It is necessary to consider the situation of families who have difficulties or anxieties about administering end-of-life care at home and seek gentle and heartwarming end-of-life care at hospitals for their loved ones, knowing that they will have to observe all the required measures against infectious diseases during their restricted visits (36). The UK has increased opportunities for bereaved institutional support, including the issuance of guidelines by the NHS to allow only one family member to visit patients who are unlikely to recover or who are days or weeks away from their death (37).

Although end-of-life care has been discussed as an issue for medical care in super-aging Japanese society, COVID-19 could be considered an opportunity to think about dying at home. As a country with one of the highest life expectancy levels, Japan has focused on care for the elderly. It is precisely for this reason that it is expected to lead the world in establishing a system of advanced end-of-life care.

## Response to Bereavement Due to COVID-19 in Japan: Lessons Learned from Past Major Disasters

Japan experienced an extremely high level of loss in the Great East Japan Earthquake of 2011 (38), which led to the development of academic and public health efforts to deal with grief; these were also deployed during the COVID-19 pandemic. For example, *the Japan Disaster Grief Support* project established after this earthquake and implemented in May 2020, provided grief-related psychoeducational materials for the bereaved (39). Musashino University, the National Center of Neurology and Psychiatry, and others developed several treatment/prevention programs for prolonged grief, which have been shown to be effective in other countries too, based on empirical evidence. These include complicated grief treatment (40) and its Japanese version, along with group cognitive behavioral therapy for the bereaved with the distress of grief less severe than complicated grief (41). Since face-to-face therapy is limited during the COVID-19 pandemic, to provide such services, it would be necessary to devise programs including web-based grief treatment and videoconferencing psychotherapy (42, 43). We provide a modified treatment program for patients in whom face-to-face treatment at an institution had to be interrupted as a preventive measure against COVID-19. Even before the COVID-19 pandemic, the U.K. and U.S. had established guidelines and training methods for telepsychological interventions and developed laws; this area has not yet been developed in Japan. In the early stages of the pandemic, some academic volunteers translated these guidelines into Japanese. The infrastructure for telepsychological interventions has been developed; however, its growth is not sufficient to meet the demand. Professionals must work together to ensure that grief support continues without interruption. To ensure that those who need help do not suffer, we must provide more flexible support, including online programs that can be implemented for bereaved families in remote areas, in preparation of the post-Corona era.

## Data Availability Statement

The original contributions presented in the study are included in the article/supplementary material, further inquiries can be directed to the corresponding author/s.

## Author Contributions

YM and YT conceived the ideas. YM developed the draft paper. MI and SN verified the draft paper as experts in grief-related research and treatment in Japan and supervised this work. YT encouraged YM to investigate the research or guidelines related to bereavement due to COVID-19 in Japan. All authors have discussed the contents and contributed to the final manuscript.

## Conflict of Interest

The authors declare that the research was conducted in the absence of any commercial or financial relationships that could be construed as a potential conflict of interest.
